# Comparative pelvic development of the axolotl (*Ambystoma mexicanum*) and the Australian lungfish (*Neoceratodus forsteri*): conservation and innovation across the fish-tetrapod transition

**DOI:** 10.1186/2041-9139-4-3

**Published:** 2013-01-23

**Authors:** Catherine Anne Boisvert, Jean MP Joss, Per E Ahlberg

**Affiliations:** 1 Australian Regenerative Medicine Institute, Monash University, Wellington Road, Clayton, VIC 3800, Australia; 2 Subdepartment of Evolution and Development, Department of Organismal Biology, Evolutionary Biology Centre, Uppsala University, Norbyvägen 18A, Uppsala 752 36, Sweden; 3 School of Biological Sciences, Macquarie University, Sydney, NSW 2010, Australia

**Keywords:** Fish-tetrapod transition, Pelvic girdle, Heterochrony, Extant phylogenetic bracketing method, Evolutionary novelty, Muscle development

## Abstract

**Background:**

The fish-tetrapod transition was one of the major events in vertebrate evolution and was enabled by many morphological changes. Although the transformation of paired fish fins into tetrapod limbs has been a major topic of study in recent years, both from paleontological and comparative developmental perspectives, the interest has focused almost exclusively on the distal part of the appendage and in particular the origin of digits. Relatively little attention has been paid to the transformation of the pelvic girdle from a small unipartite structure to a large tripartite weight-bearing structure, allowing tetrapods to rely mostly on their hindlimbs for locomotion. In order to understand how the ischium and the ilium evolved and how the acetabulum was reoriented during this transition, growth series of the Australian lungfish *Neoceratodus forsteri* and the Mexican axolotl *Ambystoma mexicanum* were cleared and stained for cartilage and bone and immunostained for skeletal muscles. In order to understand the myological developmental data, hypotheses about the homologies of pelvic muscles in adults of *Latimeria*, *Neoceratodus* and *Necturus* were formulated based on descriptions from the literature of the coelacanth (*Latimeria)*, the Australian Lungfish (*Neoceratodus)* and a salamander (*Necturus)*.

**Results:**

In the axolotl and the lungfish, the chondrification of the pelvic girdle starts at the acetabula and progresses anteriorly in the lungfish and anteriorly and posteriorly in the salamander. The ilium develops by extending dorsally to meet and connect to the sacral rib in the axolotl. Homologous muscles develop in the same order with the hypaxial musculature developing first, followed by the deep, then the superficial pelvic musculature.

**Conclusions:**

Development of the pelvic endoskeleton and musculature is very similar in *Neoceratodus* and *Ambystoma*. If the acetabulum is seen as being a fixed landmark, the evolution of the ischium only required pubic pre-chondrogenic cells to migrate posteriorly. It is hypothesized that the iliac process or ridge present in most tetrapodomorph fish is the precursor to the tetrapod ilium and that its evolution mimicked its development in modern salamanders.

## Background

**Figure 1:**
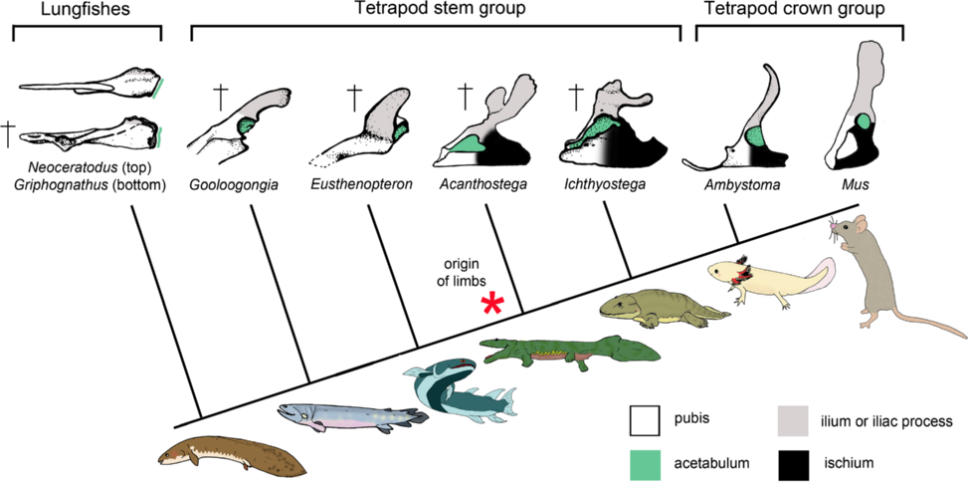
**Phylogeny spanning the fish-tetrapod transition, showing stepwise transformation of the pelvic morphology.** In lungfishes (Dipnoi) there is no iliac ramus, but a low ridge that can be homologized with the iliac process of other tetrapodomorph fishes. In the extant *Neoceratodus* the homologues of tetrapod iliac musculature attach to a low ridge anterodorsal to the acetabulum. In fish members of the tetrapod stem group (exemplified here by the rhizodont *Gooloogongia* and the osteolepiform *Eusthenopteron*), overall pelvic morphology is similar to that of lungfishes but an iliac process is present. In the stem tetrapods *Acanthostega* and *Ichthyostega* an ischium is present and overall pelvic morphology approaches that of extant salamanders such as *Ambystoma*. The boundary between ishium and pubis is approximate in stem tetrapods and axolotl as the elements are not separated by sutures. Anterior to the left. Phylogeny based on [4], *Griphognathus* and *Neoceratodus* redrawn from [15], *Gooloogongia* from [46], *Eusthenopteron* from [12], *Acanthostega* from [16], *Ichthyostega* from [17], *Ambystoma* from Figure 2, *Mus musculus* from [47]. All reconstructions by CAB.

Around 395 million years ago, the first tetrapods (four-legged vertebrates) appeared, having evolved from lobe-finned fish [1,2]. This fish-tetrapod transition was marked by many morphological transformations and ecological adaptations ranging from the evolution of fingers and toes [3-5] to new modes of respiration, hearing [6,7] and locomotion [8,9]. One of the major changes in locomotory habit is that of a shift from fish principally using their pectoral fins and lateral undulation to swim to tetrapods relying much more heavily on their hindlegs to swim and walk [10]. This shift from ‘front-wheel drive’ to ‘back wheel drive’ locomotion was enabled by the evolution of a large, weight-bearing pelvic girdle in tetrapods. In lobe-finned fishes, the pelvic girdle is composed of a crescentric pubis often connected through cartilage at the midline but lacking an ilium to connect it and, consequently, the whole fin, to the vertebral column [11]. In tetrapods, not only is an ilium present and fused to the vertebral column through a sacral rib, but an ischium is also present posterior to the pubis. The pubis and ischium from both halves of the girdle are fused along their midlines and, hence, the girdle is weight-bearing [2]. Tetrapod legs are also oriented laterally compared to the posterior orientation of fish pelvic fins. This reflects the orientation of the acetabulum, which is located on the lateral face of the pelvis in tetrapods but at the posterior end of the pelvis in fish. The morphology of Paleozoic lobe-finned fish pelves is known from descriptions of *Eusthenopteron*[12] and other fish members of the tetrapod stem group [13,14], as well as from stem dipnoans [15] and a porolepiform [11], and that of the earliest tetrapods by descriptions of *Acanthostega*[16] and *Ichthyostega*[8,17]. This provides a good picture of the general morphology on either side of the transition (Figure 1) but little information as to how the transformation occurred (Figure 1). In order to elucidate this, the pelvic girdle of the transitional fish *Panderichthys* was studied [18] but it is very fish-like and unfortunately does little to answer the following questions: Is the iliac process present in *Eusthenopteron* a precursor of the ilium of tetrapods? How did the ilium become connected to the sacral rib? How did the ischium evolve? And how did the acetabulum move during the transition?

In the absence of more informative fossils, we can use the Extant Phylogenetic Bracketing method [19] as a basis for framing hypotheses about how the transformation occurred. By comparing the development of the pelvic girdle in modern representatives of groups on either side of the transition, we can determine which aspects are similar and, thus, likely to have been conserved from fish to tetrapod, and which appear to be tetrapod innovations. This approach not only illuminates the evolution of pelvic development, but provides clues for the interpretation of the adult morphologies of transitional fossils. Heterochronies in developmental sequences have proven useful in understanding evolutionary change and are, thus, of particular interest in this context [20,21]. The Australian lungfish (*Neoceratodus forsteri*) is a morphologically conservative member of the Dipnoi and is the only lobe-finned fish available for developmental studies. Fortunately, its pelvic girdle is very similar to that of Paleozoic dipnoans [15] and a good representation of the general lobe-finned fish pelvic shape (Figure 1). The Mexican axolotl (*Ambystoma mexicanum*) is a commonly used laboratory animal, which, like other salamanders, has a pelvic morphology very similar to that of early tetrapods. In both species, a cartilaginous anterior process (pubic process in *Neoceratodus* and ypsiloid cartilage in *Ambystoma*) is present. This process is absent, or arguably unpreserved, in both Devonian lungfish and in Devonian tetrapods, and so cannot be assumed to be homologous in the two groups. However, the pubis and acetabulum are uncontroversially homologous [22] and will be used here as landmarks for the developmental comparison.

The skeletal components of the pelvic girdle do not exist in isolation, but are intimately linked both developmentally and functionally to the muscles that attach there. In order to understand the transformation of the pelvis at the fish-tetrapod transition, it is thus important to consider not just the skeleton but also the musculature. Accordingly, we present here a comparison of muscular development in *Neoceratodus* and *Ambystoma*, as a complement to the skeletal study. Establishing a robust comparative framework for the muscular data is, however, a more complicated matter than for the skeletal data because the musculature of the pelvis and hind limb is far more complex than the skeleton and the mapping of homologies between taxa correspondingly more difficult. Establishing muscle homologies has been an important focus of comparative anatomy in the 19th and early 20th century [23-26] and its importance is now being put back into focus as a way of understanding and explaining evolutionary change [27,28]. Despite the adult pelvic musculature having been described for two of the living sarcopterygians, the coelacanth *Latimeria chalumnae*[29] and the Australian lungfish *Neoceratodus forsteri*[15], as well as for several salamanders [23,24,30] and several studies having been published on hindlimb muscle homologies [25,28], no attempt has been made to establish detailed muscular homologies across the fish-tetrapod transition. We based our mapping of muscle homologies on close representatives of the taxa used in the developmental study. As a representative for the axoltol, we used the mudpuppy *Necturus maculosus*[31] because of its close phylogenetic position and similarities to *Ambystoma mexicanum*[32,33]. We present a detailed comparison of the three musculatures, developed from published descriptions, with a set of proposed homologies (Tables 1, 2, 3, 4, 5). The proposed homologies are based on the points of origin and insertion of the muscles as well as their function, following established principles for such comparisons [25,27,28].

**Table 1:** **Comparison of adductor muscles in the coelacanth *(Latimeria), *Australian lungfish *(Neoceratodus) *and mudpuppy *(Necturus)***

***Latimeria chalumnae***			***Neoceratodus forsteri***			***Necturus maculosus***		
*Superficial adductor, main bundle « Abaisseur superficiel, faiseau principal » (Ventral)*	*O:posterior border of the mesial hypophysis* *I: fascia attaching to the base of lepidotrichia*		Superficial ventromesial adductor (Dorsal and ventral)	O: Median posteroventral margin of the pelvis and the superficial ventromesial adductor from the other side (for the mesialmost fibres). I: Distal medial process on the first axial element and on radials		Ischioflexorius (Adductor) (Ventral)	O: caudal end of the ischium I: Fascia of the distal end of the shank.	
*Deep adductor « Abaisseur profond » (Ventral)*	*O: Middle of the pubis and posterior part of the pubic ramus.* *I: through tendons onto the fascia of the superficial adductor, main bundle*		Superficial ventro-lateral adductor (Ventral)	O: Median posteroventral margin of the pelvis I: Distal medial process on the first axial element and radials		Puboischio-tibialis (Adductor) (Ventral)	O: Ventral and caudal part of the pubis, most of the ischium I: Proximal end of the tibia	
*Pronators 1+2+3 (Dorsal)*	*O: very middle of the pubis on dorsal side* *I: 3 first preaxial radials and 10 first preaxial lepidotrichia*		Deep ventral adductor depressor (Dorsal)	O: Posterodorsal and posteroventral faces of the pelvis I: Base of first fin element		Ischiofemoralis (Adductor) (Ventral)	O: Ischium I:Proximal end of the femur	
*Fin adductor «Adducteur de la nageoire» (Dorsal)*	*O :Arcuate ridge on the dorsal side * *I: Fascia of the 5th pronator and base of the 9th and 10th lepidotrichia.*		Dorsomesial adductor levator (dorsal)	O: Arcuate ridge (posterior dorsal part, anterior to the acetabulum) I: Proximal and subsequent fin elements				
***Latimeria chalumnae***			***Neoceratodus forsteri***			***Necturus maculosus***		
			Mesial adductor (Dorsal)	O: Muscles of fin elements from one side I: Muscles of fin elements from the other side				
						Puboischio-femoralis externus (Adductor) (Ventral)	O: ventral surface of the girdle/Pubis and ischium I: Proximal end of the femur	

**Table 2:** **Comparison of abductor muscles in the coelacanth *(Latimeria), *Australian lungfish *(Neoceratodus) *and mudpuppy *(Necturus)***

***Latimeria chalumnae***			***Neoceratodus forsteri***			***Necturus maculosus***		
*Superficial abductor, secondary bundle « Élévateur superficiel, faiseau secondaire »*	*O: posterior internal region of the lateral hypophysis*		Superficial ventromesial abductor (Dorsal and ventral)	O: Ventrolateral face pelvis		Pubotibialis (Adductor) (Ventral)	O: lateral edge of the pubic cartilage	
	*I: Base of the 6th and 7th preaxial lepidotrichia*			I: Proximal lateral face pelvic fin			I: proximal end of the tibia	
			Superficial ventrolateral abductor (Dorsal and ventral)	O: posterior fascia of the body myotomes		Caudofemoralis (Adductor) (Ventral)	O: Caudal vertebra	
				I: Dorsolateral edge proximal axial elements of the fin			I: Proximal end femur	
Superficial abductor, main bundle « Élévateur superficiel, faiseau principal »	O: Posterior medial face of the lateral hypophysis.		Deep ventral abductor depressor (Dorsal and ventral)	O: Ventrolateral process on the pelvis		Puboischio femoralis internus (Abductor) (Ventral)	O: Internal surface of the pubic cartilage and ischium	
	I: Fascia at the base of lepidotrichia.			I: Ventral process at the distal end of the first axial element.			I: along most of the femur	
*Deep abductor “Élévateur profond”*	*O: lateral sides and base of the anterior pubic ramus I: through tendons to the fascia of the superficial abductor*		Dorsolateral abductor levator (Dorsal)	O: Swelling on the posterodorsal surface of the pelvisI: Proximal and subsequent fin elements		Iliotibialis (Dorsal surface of the thigh) (Abductor) (Dorsal)	O: base of the iliumI: extend over the knee as a tendon, inserts on the tibia	
						Ilioextensorius (Abductor) (Dorsal)	I: base of the ilium O: extend over the knee as a tendon, inserts on the tibia	
***Latimeria chalumnae***			***Neoceratodus forsteri***			***Necturus maculosus***		
*Pelvic abductor or supinator, second layer, group 5 “Supin, couche 2, groupe 5, Abducteur pelvien”*	*O: Middle of the mesial hypophysis I: following the preaxial border, inserting directly onto the six first preaxial lepidotrichia*					Iliofibularis (Abductor) (Dorsal)	O: base of the iliumI: inserts on the fibula	
						Iliofemoralis (Abductor) (Dorsal)	O: base of the iliumI: caudal edge of the femur	
								

**Table 3:** **Comparison of adductor/abductor muscles in the coelacanth *(Latimeria), *Australian lungfish *(Neoceratodus) *and mudpuppy *(Necturus)***

***Latimeria chalumnae***	***Neoceratodus forsteri***			***Necturus maculosus***		
	Radial flexors (Adductor and abductor)	O: fin elements I: fin elements		Shank flexors and shank extensors (adductor and abductor respectively)	O: Distal end of the femur	,
					I: proximal and distal end of the tibia and fibula.	

**Table 4:** **Comparison of supinator and pronator muscles in the coelacanth *(Latimeria), *Australian lungfish *(Neoceratodus) *and mudpuppy *(Necturus)***

***Latimeria chalumnae***			***Neoceratodus forsteri***			***Necturus maculosus***		
*Fourth pronator “4*^*ième*^*pronateur “ (dorsal)*	*O: posterior edge of the process on the fourth axial element I: Base of the last few preaxial lepidotrichia*		Lepidotrichial flexors (Dorsal))	O: Lateral sides of fin elements I: Lepidotrichia				
Fifth pronator “5^ième^ pronateur” (dorsal)	O: posterior edge of the process on all postaxial elements surrounding the fourth axial elementI: base of all post-axial lepidotrichia							
								
Supinator, second layer, group 3. “Supin couche 2, groupe 3”	O:Posterior edge of all four axial elements.I: Preaxial radials and preaxial lepidotrichia.		Lepidotrichial flexors (Ventral)	O: Lateral sides of fin elements I: Lepidotrichia				
								
Supinator, second layer, 4th group “Supin, couche 2, groupe 4”	O: Posterior edge of the fourth axial element. I: Postaxial lepidotrichia							
***Latimeria chalumnae***			***Neoceratodus forsteri***			***Necturus maculosus***		
Supinator, second layer, group 3 “Supin, couche 2, groupe 3”	O: Posterior edge of all four axial elements.I: Preaxial radials and preaxial lepidotrichia.		Radial-axial (Ventral and dorsal )	O: All axial elementsI : All radial elements				
Supinator, first layer. “Supin, couche1 ”	O: postaxial region between the base of the mesial hypophysis (anteriorly) and the arcuate ridge (posteriorly)I: through a tendon to the preaxial radials and preaxial lepidotrichia							
Supinator, second layer, group 2 “Supin couche 2, groupe 2 ”	O : postaxial side of the arcuate ridgeI: Preaxial radials and preaxial lepidotrichia.							
						Popliteus (pronator and supinator) (Ventral)	O: Underside of the femur near the insertion of the puboischiofemo-ralis externus)	

**Table 5:** **Comparison of hypaxial musculature in the coelacanth *(Latimeria), *Australian lungfish *(Neoceratodus) *and mudpuppy *(Necturus)***

***Latimeria chalumnae***		***Neoceratodus forsteri***	***Necturus maculosus***		
Hypaxial muscle “Muscle de l’hyposome ”	O: tip of the pubic ramus I: Hypaxial musculature	*Hypaxial muscles do not attach to the pelvic girdle*	*Rectus abdominis is not attached to the pelvic girdle*		
			Ischiocaudalis (tail flexion) (Ventral)	O: Caudal vertebrae	
				I: Caudal end of ischium	
			Caudopuboischioti-bialis (tail flexion) (Ventral)	O: Puboischiotibialis	
				I: Caudal end of ischium	

## Methods

### Salamander and lungfish larvae

Albino larvae of the Mexican axolotl (*A. mexicanum*) were purchased from the *Ambystoma* genetic stock center at the University of Kentucky, USA. They were fixed in paraformaldehyde overnight and stored in 100% methanol. The youngest larvae of the series used in this article were staged using the extended table of development developed by Nye *et al*. [34]. Given the absence of an adequate development table, older larvae were staged according to total length (in cm). Larvae of the Australian lungfish (*N. forsteri*) were raised in captivity from eggs collected in the lungfish spawning ponds at Macquarie University (protocols approved by the Macquarie University Animal Ethics Committee, approval # 2003/001). The embryos were left to hatch and develop for approximately five months, being fed on brine shrimp and bloodworms (for older larvae). The youngest larvae used in this study were staged using the developmental table developed by Kemp [35]. Fish older than stage 55 (latest stage of the table) were staged according to pelvic fin length and were given a stage number corresponding to the same developmental progress as between stages 54 and 55. The larvae were euthanized with Tricaine (MS-222) and fixed overnight in 4% paraformaldehyde with a pH of 7.4.

### Alcian blue and Alizarin red staining

#### No acid, Kimmel protocol

Lungfish were cleared and stained using a protocol without acid developed by the Kimmel Laboratory (University of Oregon, USA) for zebrafish and modified by Catherine Anne Boisvert. The larvae were eviscerated and then washed in Tris/MgCl_2_ before being transferred to Alcian stain solution (0.02% alcian in 71% EtOH and 25 mM MgCl_2_ in Tris pH 7.5 aqueous solution) for a period ranging from three to seven days. The specimens were then rehydrated through a series of ethanol in 100 mM Tris pH 7.5 and 25 mM MgCl_2_ for 30 minutes each, after which they were bleached in 3% H_2_O_2_ and 0.5% KOH for 20 to 22 hours with a change of solution. Muscles were digested away at room temperature in a solution of 1% pancreatin in 35% saturated sodium borate for 17 to 51 hours. The specimens were washed for one hour in 25% glycerol and 0.1% KOH and then stained for bone in a solution of 0.02% Alizarin stain in 10% glycerol and 0.5% KOH for two to four days. Excess stain was removed by placing them in a solution of 50% glycerol and 0.5% KOH for at least a day after which they were stored in 100% glycerol with a few crystals of thymol to avoid fungal growth. All steps were carried out on a gyrating platform at a low setting.

#### Zebrafish protocol for optical tomography

Axolotls of stages 54, 55 and 1.5 cm were cleared and stained following a protocol developed by Silke Berger from the Currie Laboratory (Australian Regenerative Medicine Institute, Monash University) for Optical Tomography on zebrafish and modified by Catherine Anne Boisvert. The larvae were eviscerated and dehydrated before being bleached in a mixture of formamide, SSC (sodium chloride citrate) and H_2_O_2_ under a light source for 20 to 25 minutes. They were then washed in PBS, dehydrated to 75% ethanol and stained in Alcian stain solution (EtOH, glacial acetic acid and 0.01 mg/ml Alcian blue) for 22 hours. The specimens were washed in 80% EtOH/Tris/MgCl_2_ and rehydrated and washed in dH_2_O. They were then stained for 11 to 24 hours in Alizarin stain (0.1 mg/ml in 0.5% KOH aqueous solution) and washed in PBS. They were then transferred to an increasing series of glycerol in PBS and stored in 100% glycerol with a few crystals of thymol. All steps were carried out on a gyrating platform at a low setting.

#### Taylor and VanDyke protocol

*Ambystoma mexicanum* specimens of sizes 2.0 cm, 2.5 cm, 3.0 cm, 3.5 cm and 4.0 cm were cleared and stained according to a protocol developed by Taylor and VanDyke [36] and modified by Catherine Anne Boisvert. The salamanders were eviscerated and washed in 80% EtOH/Tris/MgCl_2_ before being stained in Alcian blue stain (0.3 mg/ml Alcian stain in 80% EtOH and glacial acetic acid) for three days. They were neutralized in a saturated solution of sodium borate and bleached for one hour and forty minutes in a solution of 0.5% KOH and H_2_O_2_. Muscles were removed in a solution of 2.25 mg/ml trypsin in saturated sodium borate. They were then stained in Alizarin red solution (0.1 mg/ml in 0.5% KOH aqueous solution) for two to three days, rinsed in dH_2_O and transferred to an increasing series of glycerol in water. They were stored in 100% glycerol with a few crystals of thymol. All steps were carried out on a gyrating platform at a low setting.

### Immunohistochemistry

#### Klymkowsky and Hanken protocol

*Neoceratodus* larvae from stages 50 and 51 were stained as whole-mounts according to a protocol modified from Klymkowsky and Hanken [37]. The larvae were refixed overnight in Dent’s fixative and bleached for 29 hours in Dent’s bleach. The specimens were then rehydrated and washed in ‘saline cocktail’ (PBS, 0.4% Triton X-100) before being blocked in ‘serum cocktail’ (PBS, 0.4%Triton X-100, 2% bovine serum albumin (BSA), 5% dimethylsulfoxide (DMSO)) for one hour. The specimens were then incubated with the primary antibody against skeletal muscle (Hybridoma gene bank 12/101, 3.7 mg/ml IgG_1_) diluted 1:50 in ‘serum cocktail’ for five days at room temperature. They were then washed and re-blocked in ‘serum cocktail’ overnight. The larvae were then incubated in the secondary antibody (488 goat anti-mouse Alexa antibody by Molecular Probes/Invitrogen; 2 mg/ml) diluted 1:150 in ‘serum cocktail’ for two days at room temperature in the dark. They were then washed in ‘serum cocktail’ and ‘saline cocktail’ before being dehydrated to 100% methanol and transferred to an increasing series of BABB (benzyl alcohol/benzyl benzoate). All steps were carried out on a gyrating platform at a low setting.

#### Currie Laboratory protocol

The remainding stages of *Ambystoma and Neoceratodus* larvae were immunostained according to a protocol developed by Silke Berger and adapted by CAB. The pelvic region was dissected out, skinned and eviscerated. The specimens were bleached in an aqueous solution of H_2_O_2_, formamide and SSC. Specimens were permeabilized by a trypsin treatment (0.25% trypsin in PBST) and acetone cracking. Specimens were then washed in PBST and blocked for six hours in PBS containing 1% BSA and 1% DMSO. They were then incubated in the primary antibody against skeletal muscle (Hybridoma gene bank 12/101, 38 μg/ml IgG_1_) diluted 1:10, washed in PBS/BSA/DMSO and incubated in Alexa Fluor 568 goat anti-mouse IgG_1_(γ1) 2 mg/ml (Molecular Probes/Invitrogen A21124) diluted 1:150 in PBS/BSA/DMSO. They were then washed in PBS/BSA/DMSO, then in PBS over a day before being embedded in 1.5% low melting point agarose (BDH Electran, VWR 444152 g) in PBS. The blocks were left to solidify and dry at 4°C in the dark, were trimmed and slowly dehydrated to 100% methanol before being cleared through an increasing series of BABB. Most steps were carried out on a gyrating platform at a low setting.

### Imaging

All specimens were examined with a Leica MZ FLIII dissecting microscope and photographed using a Leica DFC 490 camera and the Leica Fire cam program. Fluorescent samples were examined with a mercury lamp and Leica GFP filters.

## Results

### Development of the pelvic girdle in the Australian lungfish

**Figure 2:**
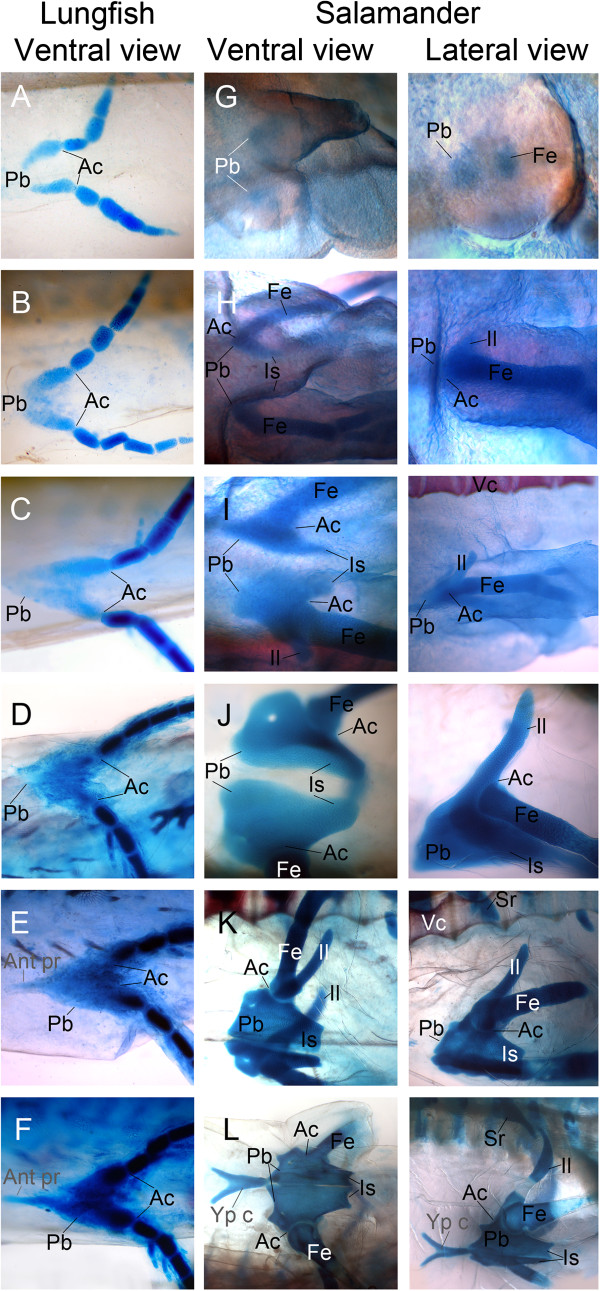
**Comparative pelvic development in the Australian lungfish (left) and the axolotl (right).** Cleared and stained larvae of *Neoceratodus forsteri* (**A-F**) and of *Ambystoma mexicanum* (**G-L**) showing development of the pelvic girdle. Cartilage is stained blue and bone in red. *Neoceratodus*: **A**) Stage 49, **B**) Stage 51, **C**) Stage 53, **D**) ‘Stage 59’, **E**) ‘Stage 60’, **F**) ‘Stage 61’ All in ventral view. *Ambystoma mexicanum*: **G**) Stage 54, **H**) 1.5 cm, **I**) 1.5 cm, **J**) 2.0 cm, **K**) 3.0 cm, **L**) 3.5 cm. Anterior to the left. The anterior process and ypsiloid cartilage are in grey to denote that they are non homologous structures. Ac, acetabulum; Ant pr, anterior process; Fe, femur; Il, ilium; Is, ischium; Pb, pubis; Sr, sacral rib; Vc, vertebral column; Yp c, ypsiloid cartilage.

Kemp’s staging table for *Neoceratodus*[35] stops at stage 55 and no staging table currently exists for older stages. Older larvae have been given a stage starting at number 56 according to general growth. These stage numbers are indicated between single quotation marks to differentiate them from the published staging table [35]. At stage 49, the pelvic girdle is already present as two narrow bands of cartilage curving mesially but not meeting at the midline (Figure 2A). In the following stages, the pelvic cartilage gradually thickens (Figure 2B). During stages 52 and 53, the pelvis gradually elongates anteriorly and the two halves start fusing at the midline (Figure 2C). During stages 54 and 55, the halves of the pelvic girdle progressively fuse anteriorly at the midline but not between the acetabula. A gap is visible between the acetabula until it closes at stage ‘56’ and thickens at stage ‘59’ (Figure 2D). At stage ‘60’, the pubic process appears as a small triangular projection much thinner than the rest of the pelvis. At stage ‘61’, the pubic process is much longer and forms the extremity of a large triangle formed by the pelvis itself (Figure 2E and F). At this stage, cartilage anterior to the acetabula is thick and a crescentric arch of strongly chondrified tissue is visible, bridging the left and right sides of the pelvic girdle. This morphology is very similar to that of a larger juvenile as described by Young *et al*. [15].

### Development of the pelvic girdle in the axolotl

Stage 54 [34] marks the beginning of pelvic development in the axolotl with the appearance of the pubis as a condensation anterior to the future acetabulum (indicated as the area between the pubic condensation and the femur) (Figure 2G). An anlage of the femur is also present at this stage posterior to the pubis condensation.(Figure 2G_lateral_). In the least developed 1.5 cm larva, the ischium has developed posterior to the pubic cartilage (Figure 2H_ventral_). The pubis is still only a small condensation of cells at the acetabulum but the ilium is now present (Figure 2H_lateral_). In another 1.5 cm larva, the pubis is larger, having extended anteriorly from the acetabulum and towards the midline as a thick condensation. The ischium is also longer, reaching farther posteriorly (Figure 2I_ventral_). The ilium is longer, projecting postero-dorsally towards the vertebral column (Figure 2I_lateral_) but not reaching it. In 2.0 cm larvae, the pubis is much larger and almost complete anteriorly (Figure 2J_ventral_). It is pierced by the obturator foramen on either side but the halves do not meet in the middle. The ischium is also larger but not complete; each half of the pelvic girdle is still triangular in shape, the anterior extremity being widest. At this stage, the ilium is much longer but far from reaching the vertebral column (Figure 2J_lateral_). Cartilage of the pubis, ischium and ilium continue to thicken and grow in 3.0 cm larvae but there is little change in overall shape. Both sides of the pelvic girdle are still unfused and roughly triangular in shape. The major difference is the development of the sacral rib and the elongation of the ilium towards it (Figure 2K). In the most developed larvae of the series (a 3.5 cm larva), the ypsiloid cartilage is complete and both sides of the pubis and ischium are almost fused (Figure 1L_ventral_). The ilium almost connects with the sacral rib and has started ossifying (white zone close to its base) but is not calcified yet, preventing the Alizarin red from binding (Figure 1L_lateral_).

**Figure 3:**
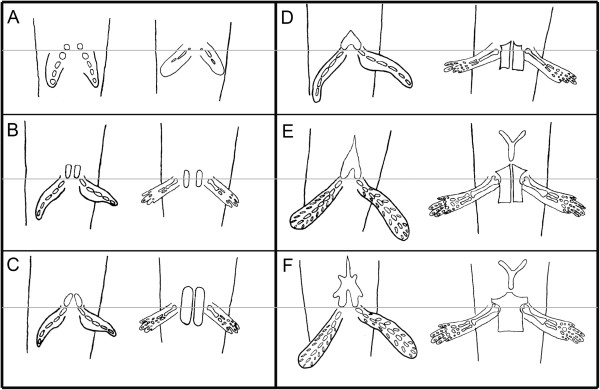
**Schematic representation of pelvic development in the Australian lungfish and the axolotl.** Each panel compares pelvic development of the Australian lungfish (left side of each panel) and the axolotl (right side). The grey line marks the position of the acetabulum. **A**) Beginning of pelvic development through cartilaginous condensations at the acetabula. **B**) The condensations extend anteriorly for the lungfish (pubis) and posteriorly (ischium) and slightly anteriorly (pubis) for the axolotl. **C**) The pubis of the lungfish continues to extend anteriorly and the pubis of the axolotl grows anteriorly. **D**) The pubis of the lungfish is now fused at the midline and is triangular shaped. The axolotl pubis is complete anteriorly and the ischium is complete posteriorly. **E**) Anterior growth of the lungfish pelvic process and appearance of the ypsiloid cartilage in the axolotl. **F**) Adult morphology of the pelvic girdle in both species. Anterior is at the top.

A schematic comparative representation of pelvic development in *Neoceratodus* and *Ambystoma*, aligned using the acetabulum as a fixed landmark, is shown in Figure 3.

#### *Comparison of the pelvic musculature in adult* Latimeria*,* Neoceratodus *and* Necturus

Table 1 displays equivalences of pelvic muscles in *Latimeria*, *Neoceratodus* and *Necturus*. Comparison of the musculature of *Neoceratodus* and *Necturus* is relatively straightforward and most muscles present in *Neoceratodus* can be associated with one or several present in *Necturus.* This is not the case for *Latimeria*, where text in italics indicates an incomplete equivalence. Often, the point of insertion is much more distal than in *Neoceratodus* or *Necturus* and it is often on lepidotrichia rather than endoskeletal fin elements. In some instances this may be the result of an incomplete dissection and it is possible that some of the muscles written in italics actually are equivalent to those of *Neoceratodus* and *Necturus* noted in plain text on the same row. However, these identifications should be regarded as tentative until a re-examination of *Latimeria* is made. For all tables, the names of the muscles were taken from their original description and have been directly translated from French for *Latimeria*.

#### Adductor muscles

All adductor muscles in *Latimeria* insert very distally on the fin, making each one an incomplete equivalent to those of *Neoceratodus* and *Necturus.* However, equivalences between *Neoceratodus* and *Necturus* are easily established. Only two muscles do not have equivalents. The mesial adductor of *Neoceratodus* was described by Young *et al*. [15] as linking muscles of both fins from either side. This is unique for *Neoceratodus*. In *Necturus,* the puboischiofemoralis externus, a superficial adductor originating from the pubis and ischium and inserting at the base of the femur, cannot be equated with any muscle present in *Neoceratodus*.

#### Abductor muscles

Abductor muscles equivalences are displayed in Table 2. Again, all muscles described in *Latimeria* insert very distally on the fin, either on lepidotrichia or on muscles inserting onto lepidotrichia. An incomplete equivalence can be established between the pelvic abductor of *Latimeria* and the iliofibularis of *Necturus* with no equivalent in *Neoceratodus*. The iliofemoralis of *Necturus* originating at the base of the ilium and inserting on the caudal edge of the femur does not have an equivalent in *Latimeria* or *Neoceratodus.*

#### Adductor/Abductor muscles

Table 3 presents muscles that either have an adductor or abductor function. The radial flexors of *Neoceratodus* can be equated to the shank flexors and extensors of *Necturus*.

#### Supinators and pronators

Many supinators and pronators have been described for *Latimeria* and most of them can be equated to lepidotrichial flexors and radial-axials present in *Neoceratodus*. Lepidotrichial flexors do not have equivalents in *Necturus* since lepidotrichia have been lost during the fin-limb transition and are, therefore, absent in all tetrapods. The radial-axials of *Neoceratodus* and their equivalents in *Latimeria* cannot be directly equated to muscles in *Necturus,* lacking pre- and post-axial radials in a ‘fish configuration’, but given that fish distal radials are precursors to digits [3,38], it is possible that those muscles were the precursors of tetrapod digit musculature. The only supinator muscle in *Latimeria* that has no equivalent in *Neoceratodus* is the supinator of the second layer, second group which originates from the pelvic girdle and inserts on preaxial radials and preaxial lepidotrichia. The popliteus of *Necturus*, wrapping around the knee, does not have direct equivalents in *Latimeria* or *Neoceratodus* since this muscle is specific to the tetrapod configuration of a knee joint.

#### Hypaxial musculature

Table 5 displays hypaxial muscles attaching to the pelvic girdle. No complete equivalences can be established despite the fact that *Latimeria*, *Neoceratodus* and *Necturus* all have a specific pelvic component of the hypaxial musculature. In *Latimeria*, the hypaxial muscle inserts onto the tip of the pubic ramus but neither the hypaxial musculature in *Neoceratodus* nor the rectus abdominis insert on the pelvic girdle. Two hypaxial muscles inserting on the ischium are present in *Necturus* but not in *Latimeria* and *Neoceratodus*. These are involved in tail movement and are specific to tetrapods in more or less elaborated ways [39].

### Development of the pelvic musculature in the Australian lungfish

**Figure 4:**
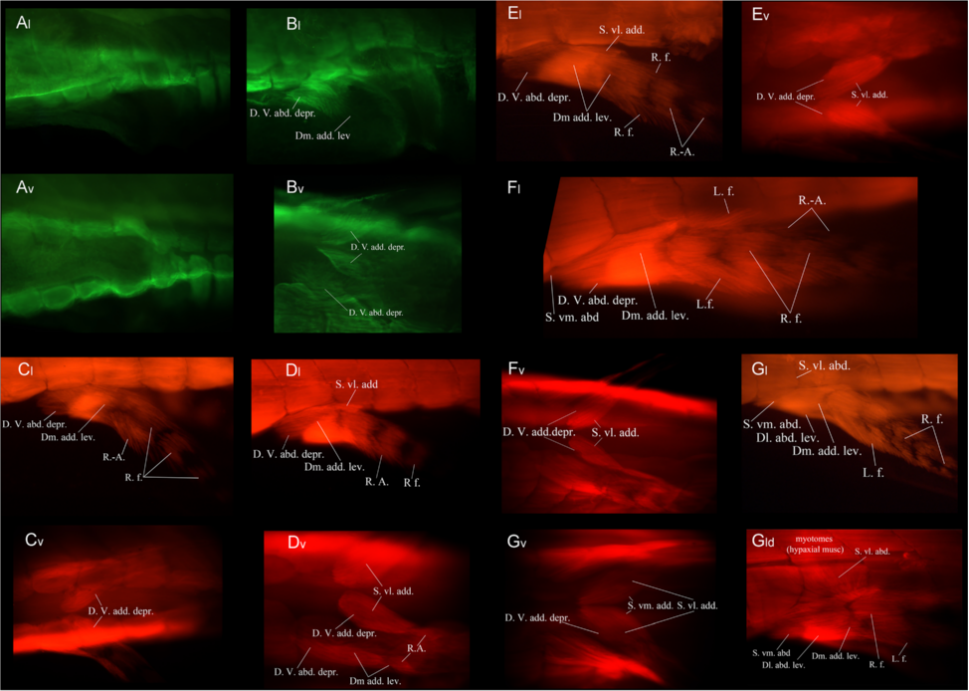
**Pelvic musculature development in the Australian lungfish.** Immunostained larvae of *Neoceratodus forsteri* showing the developing pelvic musculature. All stages were incubated in a primary antibody against skeletal muscle. **A** and **B** were visualized through a secondary anti-mouse 488 Alexa antibody and **C** and **D** were visualized with a secondary anti- IgG_1_(γ1) 568 Alexa antibody. v: ventral view and l: lateral view. **A**) Stage 50, **B**) Stage 51, **C**) Stage 52, **D**) Stage 54, **E**) Stage ‘56’, **F**) Stage ‘61’, **G**) Stage ‘63’. Dl. Abd. lev., dorsolateral abductor levator; Dm. add. lev., dorsomesial adductor levator; D. V. abd. depr., deep ventral abductor depressor; D. V. add. depr., deep ventral adductor depressor; L.f., Lepidotrichia flexors; R.-A., radial-axials; R. f., radial flexors; S. vl. abd., superficial ventrolateral abductor; S. vl. add., superficial ventrolateral adductor; S. vm. abd., superficial ventromesial abductor. Anterior to the left.

At stage 50, there is no trace of pelvic musculature. Only the hypaxial musculature is visible in lateral and ventral views (Figure 4A). At stage 51, the deep ventral abductor depressor (D. V. abd. depr.), the deep ventral adductor depressor (D. V. add. depr.) and the dorsomesial adductor levator (Dm add. lev.) appear (Figure 4B) as short and thin muscles. These muscles are longer and thicker (Figure 4C) at stage 52 and are accompanied by radial flexors (R.f.) over five or six axial elements and radial-axials (R.-A.) on the pre-axial side of the second element. Radial flexors proximal on the fin are more developed than the ones more distal and these muscles seem to develop in a proximo-distal direction following the development of axial elements. The deep ventral abductor depressor continues to develop anteriorly at stage 54 and the superficial ventrolateral adductor (S. vl. add.) is now present between the dorsal side of the pelvis and the postaxial edge of the fin (Figure 4D). No new muscles appear at stage 56. However, the dorsomesial adductor levator is more developed and its fibers extend more distally. The radial flexors are also longer, and the radial-axials extend on all six axial elements pre-axially and appear for the first time on post-axial radials (Figure 4E). At stage 61, proximal muscles (D. V. abd. depr. and Dm. add. lev.) are fully developed and begin being covered by the superficial ventromesial abductor (S. vm. abd.), which originates at a very anterior position on the pelvic girdle (Figure 4F). Radial-axials on the post-axial side of the fin are now present through its entire length. Lepidotrichial flexors (L.f.) are present through the entire pre-axial side of the fin but are only covering the second and third elements post-axially. By stage 63, all pelvic muscles have appeared except for the mesial adductor (Figure 4G). The superficial ventromesial abductor is more developed and reaches farther anteriorly onto the pelvic girdle. The dorsolateral abductor levator (Dl. Abd. lev.) can be seen as a swelling on the pre-axial side of the fin at the level of the first axial element (Figure 4G_l_). The superficial ventrolateral abductor (S. vl. abd.) is now present, visible as thin fibers reaching to the body myotomes (Figure 4G_ld_) and in ventral view, in the middle of the post-axial muscle bundles (Figure 4G_v_). Radial-axials are completely covered by radial flexors and lepidotrichial flexors, now well developed both on the pre and post-axial sides of the fin.

### Development of the pelvic musculature in the axolotl

**Figure 5:**
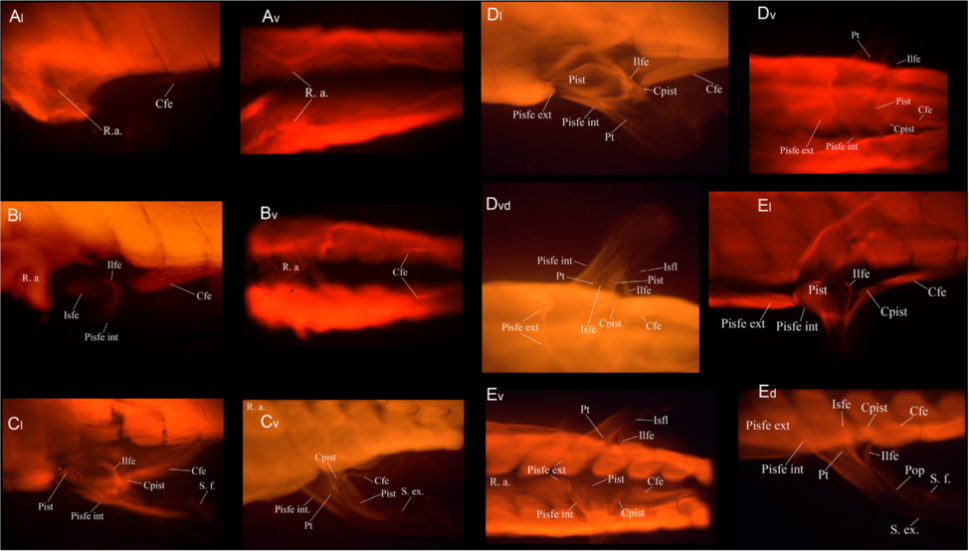
**Pelvic musculature development in the axolotl.** Immunostained larvae of *Ambystoma mexicanum* showing the developing pelvic musculature. All stages were incubated in a primary antibody against skeletal muscle and were visualized with a secondary anti-IgG_1_(γ1) 568 Alexa antibody. v, ventral view and l, lateral view; ld, lateral view, detail; d, dorsal view. **A**) Stage 55, **B**) 1.5 cm, **C**) 2.0 cm, **D**) 2.5 cm, *E) 3.0 cm. Cfe, caudofemoralis; Cpist, caudopuboischiotibialis; Ilfe, iliofemoralis; Isfe, ischiofemoralis; Isfl, ischioflexorius; Pisfe ext, puboischiofemoralis externus; Pisfe int, puboischiofemoralis internus; Pist, puboischiotibialis; Pop, popliteus; Pt, pubotibialis; R.a., rectus abdominis; S. e., shank extensors; S. f., shank flexors. Anterior to the left. *Shank extensors and flexors are absent because the distal part of the limb has been bitten of by another individual.

The very first stage of this series is at the pelvic bud stage 55 [34] when only the rectus abdominis (R. a.) and the caudofemoralis (Cfe) are present, both of which are extensions of the hypaxial musculature (Figure 5A). Those muscles flank the emerging limb bud and at the following stage (1.5 cm), the ischiofemoralis (Isfe), the puboischiofemoralis internus (Pisfe int) and the iliofemoralis are all present (Figure 5B_l_) (all muscles inserting into the limb). At 2.0 cm, the caudofemoralis muscle now extends all the way to the base of the femur and is a lot thicker. The puboischiotibialis (Pist) is now covering the ischiofemoralis and the caudopuboischiotibialis (Cpist) is now visible, linking the puboischiotibialis to bands of muscles extending towards the caudal vertebrae (Figure 5C_l_). Fibers of the iliofemoralis now extend caudally, suggesting that its point of origin on the ilium extends dorsally in connection with the dorsal extension of the iliac cartilage (Figure 2). Shank flexors (Figure 5C_l_) and extensors (Figure 5C_v_) are now present, as well as the pubotibialis (pt), visible in the middle of the thigh in the ventral view. The 2.5 cm larva is very similar to the previous stage except for the distal leg muscles, which are missing along with the rest of the leg in this individual, having fallen victim to cannibalism (a common behavior in axolotl). The puboischiotibialis is thicker and extends farther anteriorly, the iliofemoralis extends a little more dorsally and the caudopuboischiotibialis can be clearly seen connecting the puboischiotibialis to muscle fibers extending to the caudal vertebrae (Figure 5D_l_). In the ventral view, the puboischiofemoralis externus (Pisfe ext) is now present, its origin overlapping with that of the puboischiofemoralis internus (Figure 5D_v_). Due to species variation in the pelvic shape, fibers of the puboischiofemoralis externus are more parallel to the hypaxial musculature than those of *Necturus* but can be distinguished from it by its posterior point of origin. The ischioflexorius (Isfl) is probably present at this stage as a very faint band of muscle originating from the ischium and extending distally on the post-axial side of the thigh (Figure 5D_vd_). The last stage of this series is very similar to 2.5 cm. At 3.0 cm, the ischioflexorius is thicker and more visible and shank extensors and flexors as well as the popliteus are present (Figure 5E_d_). All other muscles seem to have reached their adult conformation (Figure 5E) but the ischiocaudalis, the iliotibialis, ilioextensorius and iliofibularis cannot be seen in any view. They are all small, deep muscles, which may be covered by more superficial muscles, or they might be absent altogether in *Ambystoma*.

## Discussion

### Comparative pelvic development and hypotheses of pelvic evolution

One of the major questions about the evolution of the tetrapod pelvic girdle during the fish-tetrapod transition has been the apparent shift in position of the acetabulum. In sarcopterygian fish, the fin articulation is located posteriorly on the pelvic girdle [11,12,15] whereas in early tetrapods, it is lateral [16,17] (Figure 1). This seems to imply that the acetabulum has moved across the lateral face of the pelvis during the transition. However, the fact that the acetabular region is the first part of the pelvis to develop in both *Neoceratodus* and *Ambystoma* suggests that the acetabulum should instead be regarded as a fixed landmark (grey line, Figure 3). Such a change of perspective makes the evolutionary transformation of the pelvis much easier to understand. As shown in the descriptions above, the principal difference in early pelvic development between salamander and lungfish is that in the salamander, the pubis grows anteriorly and the ischium posteriorly from the acetabular region, whereas in lungfish the pubis grows anteriorly and the ischium is absent; in other words, chondrogenic cells proliferate both anteriorly and posteriorly in the salamander, whereas in the lungfish they only proliferate anteriorly. The presence of an ischium and the lateral position (and orientation) of the acetabulum are, therefore, developmentally coupled morphological states, presumably resulting from a change in molecular signalling in the immediate ancestors to tetrapods: there is no need to postulate a 'migration' of the acetabulum. Figure 3 schematizes pelvic development in lungfish and axolotl with this hypothesis in mind. The grey line represents the position of the acetabula for both species.

Another major question about the evolution of the pelvic girdle in tetrapods concerns the origin of the ilium and its relationship, if any, to the presence of an iliac process in *Eusthenopteron, Goologongia* and other fish members of the tetrapod stem group [11,12,14,15]. The fact that the ilium of salamanders slowly extends dorsally during development, only contacting the sacral rib at a late stage when the pelvis is more or less fully formed, means that the ilium passes through a protracted developmental stage when it closely resembles the iliac process of these fishes (Figure 1). Together with the wide phylogenetic distribution of the iliac process, which suggests that it is a general character for the 'fish' part of the tetrapod stem group [11,12,14,15], this provides strong circumstantial evidence for the homology of the two structures.

### Muscle homologies in Coelacanth, Lungfish and a salamander, and the evolution of pelvic musculature

Here, the muscles of *Necturus* are described as a model for basal salamanders. There are almost twice as many muscles originating from the pelvic girdle in salamanders as in lungfish (13 versus 7) but six of these muscles are either originating or inserting on the ischium, which is a purported novelty in tetrapods. Very few pelvic muscles present in *Necturus* could not be associated with a muscle present in *Neoceratodus*. Among the exceptions is the puboischiofemoralis externus, originating mostly on the anterior process of the pubis, which is probably not homologous to the pubic process of *Neoceratodus*. However, the puboischiofemoralis is the only muscle with an ischiatic origin that could not be compared to a muscle in *Neoceratodus.* In all other cases, muscles originating from the mesial surface of the pelvic girdle in *Neoceratodus* had the same function and were very similar in terms of insertion point to muscles originating from the ischium in *Necturus*. This suggests that the ischium originated as an inflation of the posteromesial face of the pubis, created by increased proliferation of chondrogenic cells posterior to the acetabulum, an interpretation that also fits well with the data from skeletal development (Figures 2, 3). If the ischium is, in fact, a posterior projection of the pubis, the muscle groups paired up in Tables 1, 2, 3, 4 and 5 are likely to be truly homologous and the muscles themselves would not have changed substantially during the fish-tetrapod transition. This is consonant with the results from the comparative study of muscle development (see below). As for muscles originating from the ilium in *Necturus*, almost all seem to correspond to muscles originating from the posterodorsal surface of the pelvic girdle in *Neoceratodus*. Most known pelvic girdles of fossil tetrapodomorph fish have a lateral ridge or process on their posterodorsal surface [11,12,14,15]. Most of these ridges are very slight but given the fact that muscles originating on the ilium in *Necturus* originate at the very base of it, a homologous muscle could in principle originate from a small process situated at the same position on the pelvic girdle of a fish. This gives further support to the hypothesis that the iliac process of *Eusthenopteron* and other fish members of the tetrapod stem group is the precursor to the ilium of tetrapods.

### Comparative muscular development

In both *Ambystoma* and *Neoceratodus,* the hypaxial musculature develops first, followed by deep musculature originating from the pelvic girdle and inserting proximally onto the fin/limb. All muscles that have been equated in the tables above develop in the same order except for the caudofemoralis, which is the first pelvic muscle to develop in axolotls. Its homologue, the superficial ventrolateral abductor, develops last. This suggests a large heterochronic shift in the appearance of this muscle. The other, less dramatic, exception is the ischioflexorius, which develops slightly earlier in sequence than its counterpart, the superficial ventromesial adductor. The deep ventral abductor depressor, equivalent to the puboischiofemoralis internus, and the deep ventral adductor depressor, equivalent to the ischiofemoralis is the first homologous pair to appear. The dorsomesial adductor levator then appears in *Neoceratodus* and the iliofemoralis, in *Ambystoma.* These muscles have not been homologized on the basis of origin and insertion points but their developmental sequence might indicate some homology. The dorsomesial adductor levator of lungfish develops in the same sequence as the iliofemoralis and the radial-axial develop simultaneously to the caudopuboischiotibialis. Homologous pairs of slightly more superficial muscles appear next: the radial flexors/shank flexors and the superficial ventrolateral adductor/puboischiotibialis. The first of the superficial muscles to form in *Ambystoma* is the puboischiofemoralis externus, which was not homologized to any muscle in lungfish and is followed by the ischioflexorius. Its homologue (superficial ventromesial adductor) develops slightly later in lungfish, after the appearance of the superficial ventromesial abductor (synchronous with the development of the pubotibialis). This heterochronic shift might be functional in nature or an artefact of the whole mount staining method where discerning incompletely differentiated muscles can be difficult. Lepidotrichal flexors and the popliteus develop last. This might be due to the superficial nature of those muscles. Despite large differences in pelvic morphology, development of both the cartilage and muscles of these species is thus very similar. One important difference is that while the order of appearance is the same, there is more lag between consecutive muscle appearances in *Neoceratodus*. While several muscles appear at once in *Ambystoma*, new muscles appear one by one in *Neoceratodus*.

Further insight can be gained from the direction in which muscles develop. For example, the iliofemoralis is one of the first pelvic muscles to appear in 1.5 cm long *Ambystoma* larvae, the stage at which the ilium starts to extend dorsally (Figure 2I_lateral_). This muscle continues to extend dorsally, presumably following the ilium. The same thing is true for muscles attaching to the pubis: in both *Neoceratodus* and *Ambystoma*, muscles originating from the pubis extend anteriorly through development, presumably following the anterior extension of the pubic cartilage. Similarly, radial-axial and lepidotrichial flexors of the fins of *Neoceratodus* start to develop pre-axially before developing post-axially, in a manner resembling that of the appearance of radials on the fin [38]. In summary, the sequence and mode of development of the pelvic musculature appears to be substantially conserved between *Neoceratodus* and *Ambystoma*, suggesting that the morphological transformation from pelvic fin to tetrapod hind limb was accomplished without major heterochronic reorganization of muscle development.

## Conclusions

Despite large differences in pelvic morphology, the development of the pelvic girdle in *Neoceratodus* and *Ambystoma* is strikingly similar. Most pelvic muscles can be homologized between the two species and homologous muscles develop in the same order. Deep muscles develop first, followed by some muscles unique to either lungfish or salamanders and superficial muscles develop last. The only exception to this gradual development from deep to superficial is the appearance of the caudofemoralis of the axolotl at the very beginning of development whereas the superficial ventrolateral abductor of the lungfish develops last, along with the rest of the superficial musculature. The caudofemoralis originates from caudal vertebrae and is involved in tail motion. Its early appearance might be due to the very large reliance on undulatory motion of larval axolotls. Its early development might also be a consequence of the derived mode of development recently described for tetrapod pelvic appendages [40]. In lungfish, pelvic fin musculature develops from both a myotomal extension from the body wall and the migration of somite derived mesenchymal cells. However, in tetrapods, including *Ambystoma*, pelvic limb muscle formation is solely dependent on somite derived mesenchymal cells; it is argued that this mode of development would allow for earlier initiation and easier heterochronic reorganization of muscle development.

With regards to the skeleton, if we consider the acetabulum to be a fixed landmark, the evolution of the ischium simply required pre-chondrogenic cells of the pubis to migrate posteriorly as well as anteriorly in order to form it. This would explain why very similar muscles originating from the inside of the pubis in the lungfish originate from the ischium in the salamander. The ischium does not ossify in neotenic forms, such as the axolotl. However, in several salamander taxa, the ischium ossifies whilst the pubis remains unossified (Personal observation, CAB). This is reminiscent of the condition in early tetrapods [2] and might have initially been caused by the greater reliance on muscles originating from the ischium such as the puboischiotibialis. Ossification would have occurred in the ischium as a result of muscular tension which is known to promote ossification [41,42]. It would be interesting to combine the results of this study and recent biomechanical studies [8,43] to test those hypotheses. As for the origin of the ilium, some very similar muscles originating from the posterodorsal surface of the pelvis in *Neoceratodus* originate from the base of the ilium in *Ambystoma,* suggesting that the lateral ridge or process present in most tetrapodomorph fish is the precursor of the tetrapod ilium (Figure 1). This precursor would then have extended dorsally to eventually reach and articulate with the sacral rib, as observed by the development of both iliac cartilage and muscles. In order to test those hypotheses, the origin and insertion points of the muscles of *Latimeria, Neoceratodus* and *Ambystoma* should be verified using three-dimensional models generated from undissected computed tomography (CT)-scanned specimens. This will provide a more robust comparative morphological framework against which to analyze triple-stained (double immunostained for muscles and nerves plus alcian stained for cartilage) growth series of *Neoceratodus* and *Ambystoma* by optical tomography (OPT), which allows for three-dimensional three-color visualization. As a complement to these techniques for investigatng extant morphologies, the application of propagation phase contrast synchrotron microtomography (PPC-SRμCT) to the study of fossil bone histology is opening up new possibilities for detecting muscle attachments on the pelvic bones of fossil fishes and early tetrapods [44]. We expect that data extracted by these novel methods will cast a great deal of light on the transformation of the pelvic appendage at the fish-tetrapod transition and perhaps help resolve the question of the origin of tetrapod locomotion raised in several recent publications [8,43,45].

## Abbreviations

 : *Lungfish and Axolotl pelvic girdle*; Ac: Acetabulum; Ant pr: Anterior process; Fe: femur; Il: ilium; Is: ischium; Pb: pubis; Sr: Sacral rib; Vc: Vertebral column; Yp c: ypsiloid cartilage;  : *Lungfish pelvic musculature*; Dl. Abd. lev: dorsolateral abductor levator; Dm. add. lev: dorsomesial adductor levator; D. V. abd. depr: deep ventral abductor depressor; D. V. add. depr: deep ventral adductor depressor; L.f: lepidotrichia flexors; S vl abd: Superficial ventrolateral abductor; S vl add: superficial ventrolateral adductor; S. vm. abd: superficial ventromesial abductor; R.-A: radial-axials; R. f: Radial flexors;  : *Axolotl pelvic musculature*; Cfe: caudofemoralis; Cpist: caudopuboischiotibialis; Ilfe: iliofemoralis; Isfe: ischiofemoralis; Isfl: ischioflexorius; Pisfe ext: Puboischiofemoralis externus; Pisfe int: Puboischiofemoralis internus; Pist: Puboischiotibialis; Pop: Popliteus; Pt: Pubotibialis; R.a: Rectus abdominis; S. e: Shank extensors; S. f: Shank flexors.

## Competing interests

The authors declare that they have no competing interests.

## Authors’ contributions

CAB planned and performed the experiments, collected the data, prepared the manuscript and the figures. PEA contributed to the conception and design of the experiments and to the analysis and interpretation of the data. JMPJ contributed materials and all authors contributed, read and approved the final manuscript.
